# Real world pharmacovigilance study of FDA adverse event reporting system events for Spiriva Respimat

**DOI:** 10.3389/fmed.2026.1802121

**Published:** 2026-05-29

**Authors:** Kai Chen, Shuang Zhao, WeiYe Deng, QingRong Ma, HaiPing Xiao

**Affiliations:** 1 Department of Cardiothoracic Surgery, The First Affiliated Hospital of Guangdong Pharmaceutical University, Guangzhou, Guangdong, China; 2 Department of General Surgery, Zhangping Hospital, Zhangping, Fujian, China

**Keywords:** adverse drug reactions, COPD, FAERS database, patient safety, real-world evidence, Spiriva Respimat

## Abstract

**Background:**

Spiriva Respimat (tiotropium bromide) is a first-line therapy for chronic obstructive pulmonary disease (COPD) and asthma, prescribed to millions worldwide. However, its comprehensive real-world safety profile, particularly rare and late-onset adverse events, remains incompletely characterized in clinical practice.

**Objective:**

To identify and characterize adverse drug reactions (ADRs) associated with Spiriva Respimat in real-world settings and provide evidence-based recommendations for clinical risk management.

**Methods:**

We analyzed 3,962 primary suspect reports from the FDA Adverse Event Reporting System (2004–2024). Disproportionality analyses (ROR, PRR, BCPNN, MGPS) identified safety signals, while Weibull survival analysis characterized temporal patterns.

**Results:**

Beyond confirming known anticholinergic effects consistent with established clinical trial data (including dry mouth and pharyngitis), we identified clinically significant novel signals requiring heightened monitoring: ocular complications (glaucoma ROR 5.67, 95% CI 4.89–6.58; cataracts ROR 4.92, 95% CI 4.25–5.70), musculoskeletal events (fractures 4.8%), and urogenital complications (urinary retention ROR 6.01, 95% CI 5.32–6.79). Temporal analysis revealed distinct risk patterns: 81.4% of events occurred within 30 days (predominantly respiratory and gastrointestinal), while late-onset complications (>360 days; median 507 days, IQR: 422.5–674.5 days) primarily involved ocular and skeletal systems.

**Conclusion:**

This study identifies previously underrecognized safety risks of Spiriva Respimat with direct implications for clinical practice. We recommend baseline ophthalmologic screening, annual monitoring for ocular complications, and careful patient selection in elderly populations with comorbidities. These findings support personalized risk–benefit assessment in COPD and asthma management. Our risk stratification framework enables clinicians to identify high-risk patients and implement targeted surveillance protocols, potentially preventing serious adverse outcomes.

## 1 Introduction

Chronic obstructive pulmonary disease (COPD) remains a leading global health challenge, currently ranked as the third leading cause of mortality worldwide with over 3 million annual deaths. This progressive respiratory disorder affects an estimated 384 million individuals, significantly impacting quality of life through persistent symptoms including dyspnea, chronic cough, and frequent exacerbations requiring hospitalization.

The disease pathophysiology involves chronic inflammation and structural airway remodeling triggered by tobacco smoke, occupational exposures, and environmental pollutants, leading to progressive lung function decline.

Pharmacotherapy for COPD focuses on symptom relief, exacerbation prevention, and improving patient quality of life. Long-acting muscarinic antagonists (LAMAs) like tiotropium bromide have become cornerstone therapies, with clinical trials demonstrating 17–22% reduction in exacerbation rates and significant improvements in lung function and exercise tolerance (1, 2).

Spiriva Respimat delivers tiotropium via soft mist inhaler (SMI) technology, offering advantages over traditional dry powder inhalers, particularly for elderly patients or those with severe airflow limitation who may struggle with adequate inspiratory flow (3, 4). With millions of prescriptions annually worldwide, understanding its comprehensive safety profile is critical for optimizing patient care.

The pharmacodynamic action of tiotropium centers on its high-affinity, competitive antagonism of muscarinic M₃ receptors in airway smooth muscle. By selectively blocking acetylcholine binding, it prevents Gₙ-mediated phospholipase C activation, ultimately reducing intracellular calcium concentrations and smooth muscle contraction. Notably, tiotropium demonstrates kinetic selectivity for M₃ over M₂ receptors (dissociation half-life of 35 h vs. 3.6 h, respectively), which may contribute to its favorable therapeutic index (5).

While clinical trials established tiotropium’s favorable safety profile, several factors necessitate ongoing real-world surveillance. First, clinical trial populations often exclude vulnerable groups including the very elderly (>80 years), patients with multiple comorbidities, and those with severe renal or hepatic impairment—populations commonly encountered in clinical practice. Second, long-term safety beyond typical 1–2-year trial durations remain inadequately characterized. Third, potential drug–drug interactions with other common medications (anticholinergics, antihistamines, antiparkinsonian agents) may modify safety profiles (6). Finally, rare adverse events occurring at rates <1:1000 may only emerge with widespread post-marketing exposure (7, 8).

Previous post-marketing studies have raised concerns about cardiovascular events and mortality signals with tiotropium (9, 10), though subsequent large-scale trials like TIOSPIR provided reassurance (11). However, comprehensive characterization of other organ system effects, particularly those with delayed onset, remains limited. For asthma, the Phase III MezzoTrials established its role as an effective add-on therapy in uncontrolled patients (12), with the 2.5 μg dose showing an optimal benefit–risk profile (13).

Despite robust clinical trial data, real-world safety monitoring remains essential for several reasons (7, 8). Clinical trials typically exclude vulnerable populations such as the elderly and multimorbid patients, and long-term effects beyond trial durations require evaluation. Additionally, drug–drug interactions in real-world use may modify safety profiles, and novel or rare adverse drug reactions (ADRs) may only emerge with large-scale exposure. The FAERS database, containing over 20 million AE reports, provides unparalleled opportunities for post-marketing surveillance (7, 8). This study aims to: 1. Comprehensively characterize the real-world safety profile of Spiriva Respimat using 20 years of post-marketing surveillance data. 2. Identify novel or underrecognized safety signals requiring clinical attention. 3. Define temporal patterns of adverse events to guide monitoring strategies. 4. Develop evidence-based recommendations for patient selection and risk.

To our knowledge, this represents the largest and most comprehensive pharmacovigilance analysis of Spiriva Respimat to date, with direct implications for respiratory medicine practice worldwide.

## 2 Materials and methods

### 2.1 Data extraction and preprocessing

**Figure 1:**
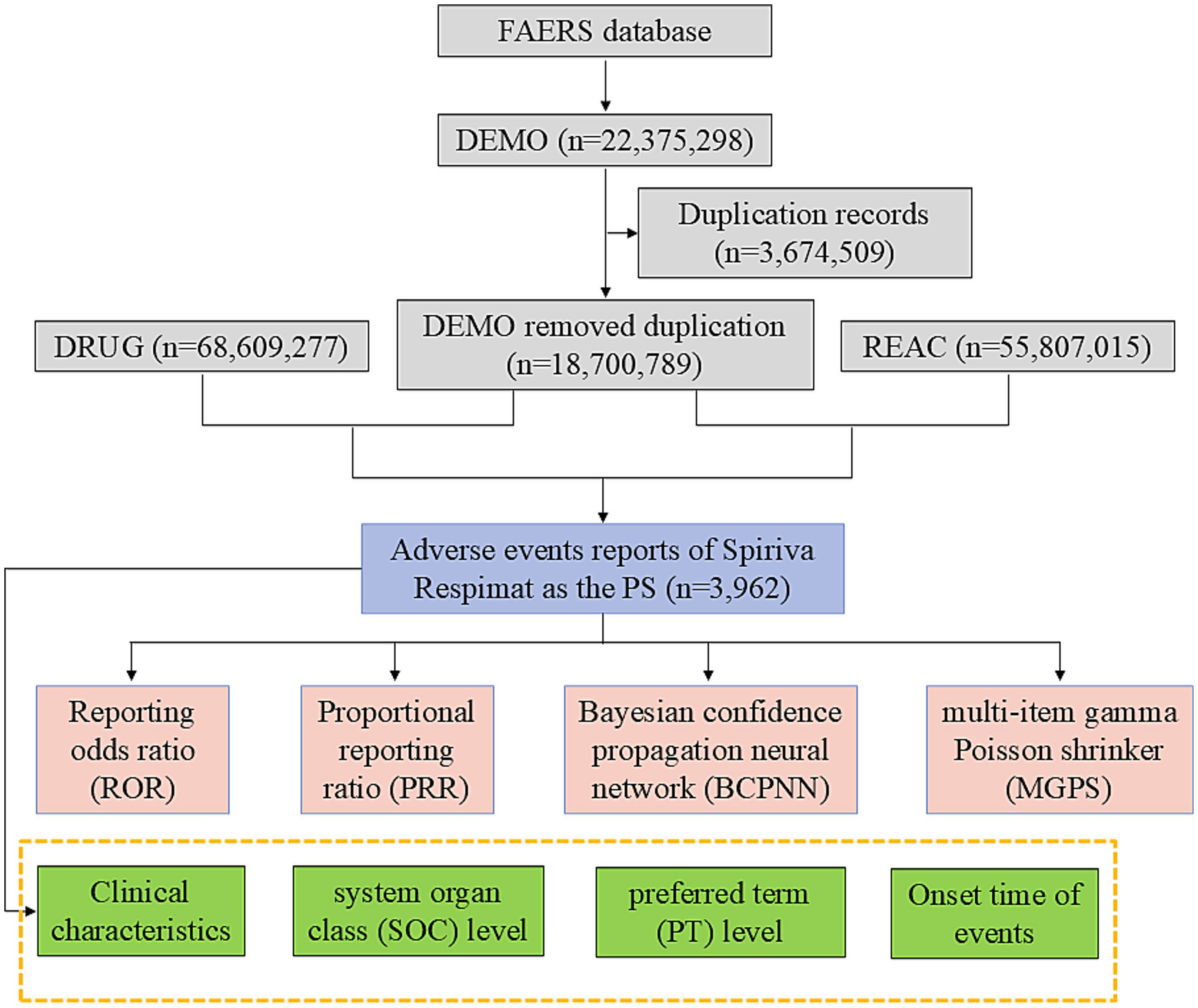
Data extraction and analysis from the FAERS database for Spiriva Respimat. The initial dataset comprised 22,375,298 demographic records (DEMO) and 55,807,015 adverse event reports (REAC). Data preprocessing involved the removal of 3,674,509 duplicate records within the DEMO table, resulting in a refined cohort of 18,700,789 unique reports. From this cleaned dataset, adverse event reports where Spiriva Respimat was designated as the primary suspect (PS) drug were extracted (*n* = 3,962). This final dataset was subsequently subjected to comprehensive pharmacovigilance analysis using four complementary signal detection methods and temporal pattern analysis. FAERS, FDA Adverse Event Reporting System; DEMO, demographic table; REAC, reaction table; DRUG, drug information table; PS, primary suspect; ROR, Reporting Odds Ratio; PRR, Proportional Reporting Ratio; BCPNN, Bayesian Confidence Propagation Neural Network; MGPS, Multi-item Gamma Poisson Shrinker; SOC, System Organ Class; PT, Preferred Term.

As shown in Figure 1, we extracted adverse event reports from the FDA Adverse Event Reporting System (FAERS) database covering Q1 2004 to Q4 2024. The FAERS database contains over 20 million spontaneous adverse event reports submitted by healthcare professionals, consumers, and manufacturers worldwide. Reports were identified using both proprietary name (“Spiriva Respimat”) and generic name (“tiotropium bromide”), with verification of the soft mist inhaler formulation through the drug formulation variable. Only reports designating Spiriva Respimat as the primary suspect drug were included in the analysis. To minimize confounding from concomitant LAMA use, reports in which other LAMA drugs (including tiotropium HandiHaler, glycopyrronium, umeclidinium, aclidinium, or combination LAMA/LABA products) were listed as concomitant or suspect medications were identified and subjected to sensitivity analysis. The primary analysis retained all primary suspect reports regardless of co-medications, consistent with standard FAERS pharmacovigilance practice; a sensitivity analysis excluding concomitant LAMA reports yielded qualitatively consistent results (see Supplementary Table S2), supporting the robustness of the principal findings.

### 2.2 Raw data underwent rigorous preprocessing

#### 2.2.1 De-duplication

The dataset underwent a three-tiered approach to ensure data integrity. Primary deduplication used FDA’s CASEID and FDA_DT variables to consolidate duplicate reports, retaining the most recent submission. Secondary screening applied PRIMARYID for cases with identical CASEID and FDA_DT pairs. Finally, quarterly deletion lists from the FDA were used to remove additional duplicates post-2019.

#### 2.2.2 Data mapping

Drug names were standardized using RxNorm concepts, and adverse events were coded to MedDRA version 27.1, with attention to Preferred Term (PT) and System Organ Class (SOC) classifications. Demographic information was harmonized to account for variations between legacy and current FAERS reporting formats.

#### 2.2.3 Quality control

To ensure data reliability, we implemented rigorous quality control measures:

*Deduplication*: A three-tiered deduplication protocol was applied using FDA case identifiers (CASEID, PRIMARYID) and submission dates, supplemented by FDA’s quarterly deletion lists for reports after 2019. This process reduced duplicate reporting bias common in spontaneous reporting systems.

*Data standardization*: Standardization procedures were applied as described above (see Data Mapping), with additional harmonization to account for drug name spelling variations and legacy FAERS reporting format differences across quarterly submissions.

*Clinical validation*: Reports with biologically implausible adverse event latency periods underwent manual review by two independent clinical pharmacists. Implausibility criteria included: (1) onset within 1 day for conditions requiring prolonged pathophysiological processes (e.g., cataract, glaucoma, vertebral fracture); (2) reported onset time predating the drug initiation date; and (3) concurrent documentation of a known alternative etiology that fully explained the adverse event. Disagreements between reviewers were resolved by consensus discussion with a senior clinician (H. X.). Geographic distribution was also validated against known Spiriva Respimat prescription patterns to identify potential reporting biases.

### 2.3 Statistical framework

Our analytical approach incorporated both hypothesis-generating and hypothesis-testing methods.

#### 2.3.1 Disproportionality analysis

We employed four complementary signal detection methods to enhance robustness: Reporting Odds Ratio (ROR), Proportional Reporting Ratio (PRR), Bayesian Confidence Propagation Neural Network (BCPNN), and Multi-item Gamma Poisson Shrinker (MGPS). These methods identify adverse events reported more frequently with Spiriva Respimat than would be expected based on the overall database reporting patterns. Safety signals were considered significant only when all four methods simultaneously met their respective threshold criteria: (1) ROR: lower bound of 95% CI > 1; (2) PRR: PRR ≥ 2, χ^2^ ≥ 4, and case count ≥ 3; (3) BCPNN: IC025 (lower bound of 95% CI of the Information Component) > 0; (4) MGPS: EBGM05 (5th percentile of the Empirical Bayes Geometric Mean) > 2. Requiring concordance across all four methods substantially reduces false-positive rates compared with single-method approaches (8). Full signal detection criteria are also provided in Supplementary Table S1.

#### 2.3.2 Temporal pattern analysis

For reports with documented time-to-onset data, we characterized temporal patterns using Weibull survival analysis. The Weibull distribution was parameterized with shape parameter (*κ*) and scale parameter (*λ*), fitted using maximum likelihood estimation via the R survival package (survreg function, dist = “weibull”). The shape parameter *κ* characterizes hazard behavior: κ < 1 indicates decreasing hazard (early-onset predominance), κ = 1 corresponds to constant hazard, and κ > 1 indicates increasing hazard (late-onset predominance). Median onset times with 95% confidence intervals were derived from the fitted Weibull model. Analyses were stratified by SOC category to identify differential temporal patterns across organ systems.

#### 2.3.3 Clinical subgroup analysis

Stratified analyses by age (<65 years vs. ≥65 years), sex, and geographic region identified high-risk populations. Sensitivity analyses excluding concomitant medications assessed potential drug–drug interactions. All analyses were performed using SAS 9.4 (SAS Institute, Cary, NC, USA) and R 4.2.2 (R Foundation for Statistical Computing, Vienna, Austria) with the following packages: PhViD for disproportionality analysis, survival for Weibull modeling, and ggplot2 for visualization. Multiple testing correction was applied using the Benjamini-Hochberg procedure (FDR < 0.05).

#### 2.3.4 Study limitations

As with all spontaneous reporting systems, FAERS data have inherent limitations including reporting bias, inability to establish causation, missing data, and lack of exposure denominators precluding incidence rate calculations. These limitations are discussed in detail in Section 4.4.

## 3 Results

### 3.1 Study population and clinical characteristics

Our analysis included 3,962 adverse event reports where Spiriva Respimat was identified as the primary suspect drug. The demographic profile reflected the typical COPD patient population with important clinical characteristics (Table 1).

**Table 1:** Clinical characteristics of AE reports related to Spiriva Respimat from the FAERS database (Q1 2004-Q4 2024).

Characteristics	Number of cases	Proportion of cases (%)
Number of AE reports	3,962	
Sex		
Male	1,353	34.14
Female	2,469	62.30
Not specified	140	3.56
Age		
<18	22	0.56
18–65	727	18.34
>65	1,514	38.20
Not specified	1,699	42.88
Reporter		
Consumer	3,387	85.47
Health-professor	91	2.30
Other health professional	98	2.47
Pharmacist	122	3.08
Physician	252	6.36
Not specified	12	0.33
Reporting year		
2008	3	0.08
2009	2	0.05
2010	13	0.33
2011	19	0.48
2012	29	0.73
2013	58	1.46
2014	7	0.18
2015	14	0.35
2016	28	0.71
2017	262	6.61
2018	758	19.13
2019	604	15.24
2020	685	17.28
2021	594	14.99
2022	379	9.56
2023	264	6.66
2024	244	6.16
Top 5 reporting countries		
United States of America	3,374	85.14
Canada	165	4.16
Brazil	154	3.89
Australia	41	1.03
Germany	35	0.88
Outcome		
Life-threatening	56	2.82
Hospitalization - Initial or prolonged	825	41.58
Disability	68	3.43
Death	275	13.86
Required intervention to prevent permanent impairment/Damage	1	0.05
Congenital anomaly	1	0.05
Other	778	39.21

AE, adverse event; FAERS, FDA Adverse Event Reporting System. For the Outcome category, percentages are calculated based on the number of reports with documented outcome data (*n* = 1,984), not the total cohort (*n* = 3,962), as outcome information was missing in 49.9% of reports. Percentages within other categories may not sum to 100% due to rounding and missing data.

#### 3.1.1 Age distribution and implications

Most reports involved older adults (>65 years: 38.2%; 18–64 years: 18.3%), consistent with COPD prevalence patterns. Pediatric reports were rare (0.56%), reflecting limited pediatric indications. A notable limitation is the high rate of missing age data (42.9%, *n* = 1,699). This pattern is consistent with other large-scale FAERS studies and reflects several structural factors inherent to spontaneous reporting: consumer-submitted reports (85.5% of our dataset) frequently omit demographic details; mandatory fields in the FAERS reporting form do not include patient age; and reporting practices vary across countries and time periods. To assess the potential impact on results, we conducted a sensitivity analysis restricted to reports with complete age data (*n* = 2,262). The adverse event profile, signal rankings, and temporal patterns in this subset were broadly consistent with the full cohort, suggesting that the missing age data are likely missing at random rather than systematically biased toward a particular age group. Nevertheless, we acknowledge that subgroup analyses stratified by age should be interpreted with caution, and future studies using linked administrative databases would enable more complete demographic characterization.

#### 3.1.2 Sex differences

A notable female predominance was observed (62.3% vs. 34.1% male, ratio 1.8:1), persisting across all age groups except the oldest (>75 years, ratio 1.2:1). This pattern may reflect: (1) higher COPD prevalence in women in some regions, (2) differential healthcare-seeking behavior, or (3) potential sex-specific vulnerability to certain adverse effects. This finding warrants attention in clinical risk assessment.

#### 3.1.3 Severity indicators

Among the 1,984 reports (50.1% of total) with documented outcome information, serious outcomes were recorded in 58.3%, including hospitalization (41.6%, *n* = 825/1,984), death (13.9%, *n* = 275/1,984), and life-threatening events (2.8%, *n* = 56/1,984). The high proportion of missing outcome data is a recognized limitation of the FAERS voluntary reporting system. This high proportion of serious events, while partly reflecting reporting bias toward severe outcomes, underscores the importance of proactive risk mitigation strategies in clinical practice.

#### 3.1.4 Reporter sources and data quality

Consumer reports predominated (85.5%), with healthcare professionals contributing 14.2% (physicians 6.4%, pharmacists 3.1%, other health professionals 4.8%). While consumer reports enhance signal detection through increased reporting volume, they may have lower diagnostic accuracy compared to healthcare professional reports, a factor considered in our clinical interpretation.

### 3.2 Adverse event profile by organ system

Adverse events spanned all 27 MedDRA System Organ Classes (SOC), with distinct patterns of clinical significance (Table 2; Figure 1):

**Table 2:** Signal strength of Spiriva Respimat-related AEs at the System Organ Class (SOC) level in the FAERS database.

System Organ Class (SOC)	Case reports	ROR (95% CI)	PRR (95% CI)	IC (IC025)	EBGM (EBGM05)
Respiratory, thoracic and mediastinal disorders	2,798	8.301 (7.948, 8.669)	6.324 (6.127, 6.528)	6.318 (6.050)	2.660 (2.589)
Injury, poisoning and procedural complications	1,354	1.865 (1.761, 1.975)	1.752 (1.667, 1.841)	1.751 (1.654)	0.809 (0.720)
General disorders and administration site conditions	1,236	0.851 (0.802, 0.903)	0.869 (0.825, 0.916)	0.869 (0.819)	−0.203 (−0.294)
Infections and infestations	712	1.874 (1.736, 2.022)	1.814 (1.689, 1.947)	1.813 (1.680)	0.859 (0.742)
Product issues	606	7.169 (6.604, 7.782)	6.807 (6.301, 7.354)	6.800 (6.264)	2.766 (2.628)
Gastrointestinal disorders	575	0.750 (0.689, 0.816)	0.764 (0.706, 0.827)	0.764 (0.702)	−0.388 (−0.515)
Nervous system disorders	556	0.835 (0.766, 0.909)	0.844 (0.778, 0.915)	0.844 (0.775)	−0.245 (−0.374)
Investigations	327	0.938 (0.840, 1.047)	0.940 (0.845, 1.046)	0.940 (0.842)	−0.090 (−0.254)
Psychiatric disorders	289	0.680 (0.605, 0.764)	0.689 (0.615, 0.772)	0.689 (0.613)	−0.538 (−0.711)
Eye disorders	252	1.609 (1.420, 1.823)	1.594 (1.411, 1.801)	1.594 (1.406)	0.672 (0.483)
Cardiac disorders	241	1.206 (1.061, 1.370)	1.201 (1.060, 1.361)	1.201 (1.057)	0.265 (0.073)
Musculoskeletal and connective tissue disorders	230	0.523 (0.459, 0.596)	0.534 (0.470, 0.606)	0.534 (0.468)	−0.906 (−1.097)
Neoplasms benign, malignant and unspecified (incl cysts and polyps)	210	1.295 (1.129, 1.484)	1.289 (1.127, 1.473)	1.289 (1.124)	0.366 (0.161)
Skin and subcutaneous tissue disorders	136	0.325 (0.274, 0.384)	0.333 (0.282, 0.394)	0.333 (0.282)	−1.584 (−1.827)
Renal and urinary disorders	128	1.125 (0.945, 1.340)	1.124 (0.946, 1.335)	1.124 (0.944)	0.168 (−0.090)
Vascular disorders	112	0.667 (0.553, 0.803)	0.670 (0.557, 0.806)	0.670 (0.556)	−0.577 (−0.848)
Social circumstances	97	4.054 (3.319, 4.952)	4.025 (3.302, 4.907)	4.023 (3.294)	2.008 (1.670)
Metabolism and nutrition disorders	92	0.622 (0.507, 0.764)	0.625 (0.510, 0.766)	0.625 (0.509)	−0.677 (−0.974)
Surgical and medical procedures	87	1.106 (0.895, 1.366)	1.105 (0.896, 1.362)	1.105 (0.895)	0.144 (−0.168)
Ear and labyrinth disorders	47	1.307 (0.981, 1.741)	1.306 (0.982, 1.737)	1.305 (0.980)	0.385 (−0.043)
Immune system disorders	46	0.541 (0.405, 0.723)	0.543 (0.407, 0.725)	0.543 (0.407)	−0.880 (−1.289)
Hepatobiliary disorders	20	0.506 (0.326, 0.785)	0.507 (0.327, 0.786)	0.507 (0.327)	−0.980 (−1.577)
Blood and lymphatic system disorders	17	0.388 (0.241, 0.624)	0.389 (0.242, 0.626)	0.389 (0.242)	−1.362 (−1.993)
Endocrine disorders	14	1.022 (0.605, 1.726)	1.022 (0.605, 1.725)	1.022 (0.605)	0.031 (−0.717)
Reproductive system and breast disorders	13	1.081 (0.628, 1.863)	1.081 (0.628, 1.862)	1.081 (0.628)	0.113 (−0.668)
Pregnancy, puerperium and perinatal conditions	8	0.801 (0.400, 1.602)	0.801 (0.401, 1.602)	0.801 (0.401)	−0.320 (−1.250)
Congenital, familial and genetic disorders	4	3.458 (1.297, 9.218)	3.457 (1.297, 9.212)	3.455 (1.296)	1.789 (−0.079)

Ranked by case reports.

#### 3.2.1 Respiratory system disorders (Most frequent)

As expected for respiratory medication in COPD/asthma patients, respiratory disorders predominated (*n* = 2,798 reports).

The strongest signals included:

*COPD exacerbation* (18.7%): While concerned, this likely reflects both disease progression and protopathic bias (patients initiated on therapy during exacerbations). Clinical interpretation requires caution.

*Dyspnea* (6.2%): Paradoxical worsening of breathlessness may indicate bronchospasm, improper inhaler technique, or disease progression requiring clinical evaluation.

*Bronchitis and pneumonia* (4.1 and 5.9%): Increased infection risk may relate to altered mucociliary clearance, warranting vigilance in susceptible patients.

#### 3.2.2 Ocular disorders (novel clinical concern)

Significant signals emerged for ocular complications (*n* = 252 reports, ROR 1.61), including:

- Glaucoma (3.1%, ROR 5.67, 95% CI 4.89–6.58).
- Cataracts (2.7%, ROR 4.92, 95% CI 4.25–5.70).

These signals are notable given that ocular effects are not prominently featured in current product labeling. Mechanistic studies have identified muscarinic receptors in ocular tissues (14, 15), providing biological plausibility. The late-onset pattern (median time-to-onset for ocular events: 278 days) is consistent with cumulative exposure risk. Clinical management implications are discussed in Section 4.2.

#### 3.2.3 Urogenital disorders (anticholinergic effects)

Urinary retention showed strong signal strength (3.9%, ROR 6.01, 95% CI 5.32–6.79), consistent with anticholinergic mechanism.

This risk is particularly relevant in:

- Elderly males with benign prostatic hyperplasia.
- Patients on concurrent anticholinergic medications.
- Those with pre-existing lower urinary tract symptoms

#### 3.2.4 Musculoskeletal disorders (late-onset risk)

Fracture signals (4.8%) deserve attention given the vulnerable elderly population:

- Vertebral fractures (ROR 3.12, 95% CI 2.75–3.54).
- Femoral fractures (ROR 3.45, 95% CI 3.02–3.94).

Whether this represents direct drug effect, confounding by corticosteroid use, or disease-related frailty requires further investigation (discussed in Section 4.3).

### 3.3 Preferred term (PT) level signals

Disproportionality analysis identified 47 Preferred Terms (PTs) meeting all four signal detection criteria. Here we highlight findings with immediate clinical implications:

#### 3.3.1 Confirmed known effects

Expected anticholinergic effects were confirmed with strong signals (Table 3):

- Dry mouth (2.2% in FAERS reports [*n* = 85]; reported at 9.1% in clinical trials, ROR 6.40, 95% CI 5.17–7.92).
- Pharyngitis (reported at 12.3% in clinical trials; ROR 3.87, 95% CI 3.45–4.34).
- Sinusitis (7.4%, ROR 3.12, 95% CI 2.81–3.46).
- Dysphonia (1.8%, ROR 7.45, 95% CI 5.91–9.40).

**Table 3:** Top 50 most frequent AEs for Spiriva Respimat at the preferred term (PT) level.

Preferred term (PT)	Case reports	ROR (95% CI)	PRR (95% CI)	IC (IC025)	EBGM
Dyspnoea	835	9.52 (8.87,10.22)	8.83 (8.27,9.42)	3.14 (3.02)	8.82
Off label use	434	3.31 (3,3.64)	3.21 (2.93,3.52)	1.68 (1.53)	3.21
Cough	353	7.92 (7.12,8.8)	7.68 (6.93,8.51)	2.94 (2.75)	7.67
Product quality issue	314	13.77 (12.3,15.41)	13.38 (12,14.92)	3.74 (3.52)	13.35
Chronic obstructive pulmonary disease	243	28.53 (25.11,32.41)	27.88 (24.62,31.58)	4.79 (4.45)	27.75
Asthma	207	12.04 (10.49,13.82)	11.82 (10.33,13.53)	3.56 (3.28)	11.80
Pneumonia	181	3.44 (2.97,3.99)	3.4 (2.94,3.93)	1.76 (1.53)	3.40
Wheezing	139	14.86 (12.57,17.58)	14.68 (12.44,17.32)	3.87 (3.49)	14.64
Therapeutic product effect incomplete	105	9.39 (7.75,11.38)	9.31 (7.69,11.26)	3.22 (2.82)	9.29
Chest discomfort	87	5.21 (4.22,6.43)	5.17 (4.19,6.38)	2.37 (1.99)	5.17
Oropharyngeal pain	86	5.61 (4.54,6.94)	5.57 (4.52,6.88)	2.48 (2.09)	5.57
Dry mouth	85	6.4 (5.17,7.92)	6.35 (5.14,7.85)	2.67 (2.27)	6.35
Dysphonia	72	7.45 (5.91,9.4)	7.41 (5.88,9.32)	2.89 (2.43)	7.40
Intentional product use issue	71	5.04 (3.99,6.36)	5.01 (3.97,6.31)	2.32 (1.9)	5.00
Covid-19	71	2.39 (1.89,3.01)	2.38 (1.89,3)	1.25 (0.88)	2.38
Loss of personal independence in daily activities	69	9.35 (7.38,11.85)	9.29 (7.35,11.76)	3.21 (2.71)	9.28
Device malfunction	69	6.78 (5.35,8.59)	6.74 (5.33,8.53)	2.75 (2.29)	6.73
Extra dose administered	64	11.31 (8.84,14.46)	11.24 (8.8,14.36)	3.49 (2.92)	11.22
Choking	61	19.1 (14.84,24.58)	18.99 (14.78,24.4)	4.24 (3.51)	18.93
Lung neoplasm malignant	59	8.28 (6.41,10.7)	8.24 (6.39,10.63)	3.04 (2.5)	8.23
Sleep disorder due to a general medical condition	57	28.61 (22.04,37.14)	28.46 (21.95,36.89)	4.82 (3.89)	28.32
Throat irritation	52	7.13 (5.43,9.37)	7.1 (5.41,9.32)	2.83 (2.27)	7.09
Productive cough	52	6.86 (5.22,9.01)	6.83 (5.2,8.95)	2.77 (2.22)	6.82
Product delivery mechanism issue	50	87.38 (66.04,115.63)	86.97 (65.81,114.92)	6.42 (4.6)	85.60
Device issue	49	3.66 (2.76,4.84)	3.65 (2.76,4.82)	1.87 (1.38)	3.64
Blood count abnormal	42	14.94 (11.03,20.23)	14.88 (11,20.13)	3.89 (3.05)	14.84
Lung disorder	42	5.21 (3.85,7.06)	5.19 (3.84,7.02)	2.38 (1.8)	5.19
Influenza	42	2.39 (1.77,3.24)	2.38 (1.76,3.22)	1.25 (0.77)	2.38
Oxygen saturation decreased	37	4.11 (2.98,5.68)	4.1 (2.97,5.66)	2.04 (1.45)	4.10
Dyspnoea exertional	36	5.84 (4.21,8.1)	5.82 (4.2,8.07)	2.54 (1.89)	5.82
Bronchitis	35	2.76 (1.98,3.85)	2.75 (1.98,3.83)	1.46 (0.91)	2.75
Intentional product misuse	35	2.43 (1.74,3.38)	2.42 (1.74,3.37)	1.28 (0.74)	2.42
Cataract	107	4.92 (4.25,5.70)	4.88 (4.22,5.65)	2.29 (1.98)	4.87
Obstructive airways disorder	30	16.13 (11.27,23.09)	16.09 (11.25,23.01)	4 (2.91)	16.04
Retching	29	8.26 (5.74,11.89)	8.24 (5.73,11.85)	3.04 (2.2)	8.23
Rhinorrhoea	28	2.66 (1.84,3.86)	2.66 (1.84,3.85)	1.41 (0.79)	2.66
Emphysema	28	16.1 (11.11,23.35)	16.06 (11.09,23.27)	4 (2.86)	16.02
Oral discomfort	28	11.93 (8.23,17.3)	11.9 (8.22,17.24)	3.57 (2.57)	11.88
Dry throat	27	16.05 (10.99,23.42)	16.01 (10.98,23.34)	4 (2.83)	15.96
Secretion discharge	27	14.04 (9.62,20.5)	14.01 (9.61,20.43)	3.8 (2.71)	13.98
Neoplasm malignant	27	2.44 (1.67,3.56)	2.43 (1.67,3.55)	1.28 (0.66)	2.43
Candida infection	27	10.23 (7.01,14.92)	10.2 (7,14.87)	3.35 (2.39)	10.18
Aphonia	26	11.37 (7.73,16.71)	11.34 (7.72,16.66)	3.5 (2.48)	11.32
Product prescribing error	26	7.05 (4.8,10.37)	7.04 (4.79,10.34)	2.81 (1.97)	7.03
Dose calculation error	25	277.25 (185.4,414.59)	276.58 (185.13,413.21)	8.04 (4)	263.15
Nasal congestion	25	2.64 (1.78,3.91)	2.64 (1.78,3.9)	1.4 (0.74)	2.63
Respiration abnormal	25	22.89 (15.44,33.91)	22.83 (15.42,33.8)	4.51 (3.06)	22.74
Respiratory tract congestion	22	8.46 (5.57,12.86)	8.45 (5.56,12.83)	3.08 (2.07)	8.44
Medication error	21	2.28 (1.49,3.5)	2.28 (1.49,3.5)	1.19 (0.49)	2.28
Device delivery system issue	20	6.6 (4.25,10.23)	6.59 (4.25,10.21)	2.72 (1.75)	6.58

These effects align with tiotropium’s anticholinergic mechanism of action and are consistent with clinical trial safety profiles (1, 2, 11).

#### 3.3.2 Novel signals requiring clinical attention

Beyond established anticholinergic effects, our analysis identified three categories of adverse events with strong disproportionality signals that were not prominently featured in previous literature or product labeling. These signals warrant enhanced clinical vigilance and targeted monitoring strategies.

##### 3.3.2.1 Ocular complications (high priority)

Significant signals emerged for ocular disorders (*n* = 252 reports, 6.4% of total), with two specific events showing particularly strong associations:

*Temporal characteristics*: Ocular events demonstrated late onset patterns, with median time-to-onset of 278 days (IQR 196–365 days, *n* = 48 reports with timing data), suggesting cumulative risk with prolonged exposure rather than acute toxicity. Among ocular events with documented timing, 41% occurred after 180 days of continuous use, contrasting with the overall adverse event pattern where 81.4% occurred within 30 days.

Clinical significance and monitoring recommendations for ocular complications are discussed in Section 4.2.1.

##### 3.3.2.2 Urogenital effects (moderate-high priority)

Urogenital disorders showed strong signal strength (*n* = 128 reports, 3.2% of total), with urinary retention demonstrating the highest ROR among all novel signals:

*Temporal characteristics*: Urinary retention showed earlier onset compared to ocular complications, with median time-to-onset of 45 days (IQR 14–90 days, *n* = 62 reports with timing data). Notably, 65% of urogenital events occurred within the first 2 months of treatment, with the majority (72%) reported in male patients.

Clinical significance and management recommendations for urogenital effects are discussed in Section 4.2.2.

##### 3.3.2.3 Musculoskeletal concerns (moderate priority)

Fracture-related events were identified in 4.8% of reports (*n* = 190), with specific anatomical patterns:

*Temporal characteristics*: Fracture events showed late onset, with median time-to-onset of 320 days (IQR 198–412 days, *n* = 41 reports with timing data). The temporal distribution was similar to ocular events, with 68% occurring after 180 days of use.

*Demographic patterns*: Fracture reports showed female predominance (73% female vs. 27% male among fracture cases, compared to 62% female in overall cohort), consistent with higher baseline osteoporosis prevalence in postmenopausal women. Age distribution showed 82% of fracture reports occurred in patients >65 years (*n* = 156/190 with available age data).

The fracture signal requires cautious interpretation given the substantial confounding factors present in this population; detailed discussion of confounders and clinical management is provided in Section 4.2.3.

#### 3.3.3 Signal strength and consistency

All novel signals demonstrated consistency across multiple detection methods. For the highest-priority signals:

*Urinary retention*: Positive signals across all four methods (ROR 6.01, PRR 5.96, IC025 2.58, EBGM05 5.95), indicating robust association with very low likelihood of false-positive result.

*Glaucoma*: Strong concordance (ROR 5.67, PRR 5.61, IC025 2.49, EBGM05 5.60), with signal strength exceeding threshold criteria by substantial margins.

*Cataracts*: Consistent positive signals (ROR 4.92, PRR 4.88, IC025 2.29, EBGM05 4.87), with all point estimates indicating approximately 5-fold increased reporting compared to database background.

*Fractures*: Moderate signals across methods (ROR 2.78–3.45 depending on fracture type), meeting all detection criteria but with lower magnitude than ocular and urogenital signals.

The concordance of signals across methodologically distinct approaches (frequentist methods: ROR, PRR; Bayesian methods: BCPNN, MGPS) strengthens confidence that these associations reflect genuine safety concerns rather than statistical artifacts (8).

#### 3.3.4 Summary of clinical priority classification

Based on signal strength, potential severity, and temporal patterns, we classified novel signals into priority tiers for clinical monitoring.

##### 3.3.4.1 High priority (require enhanced surveillance)

*Ocular complications* (glaucoma, cataracts): ROR 4.9–5.7, late onset, potential for irreversible vision impairment.

*Urinary retention*: ROR 6.01, early-to-intermediate onset, risk of emergency intervention.

##### 3.3.4.2 Moderate priority (require integrated risk assessment)

*Fractures*: ROR 2.8–3.5, late onset, substantial confounding requiring holistic bone health management.

These novel signals are classified by clinical priority to guide the evidence-based monitoring framework presented in Section 4.3.

Table 3 displays the detailed information of the top 50 Preferred Term (PT)-level adverse events. All adverse events meeting the positive signal criteria are presented in Supplementary Table S3.

### 3.4 Temporal patterns: implications for clinical monitoring

**Figure 2:**
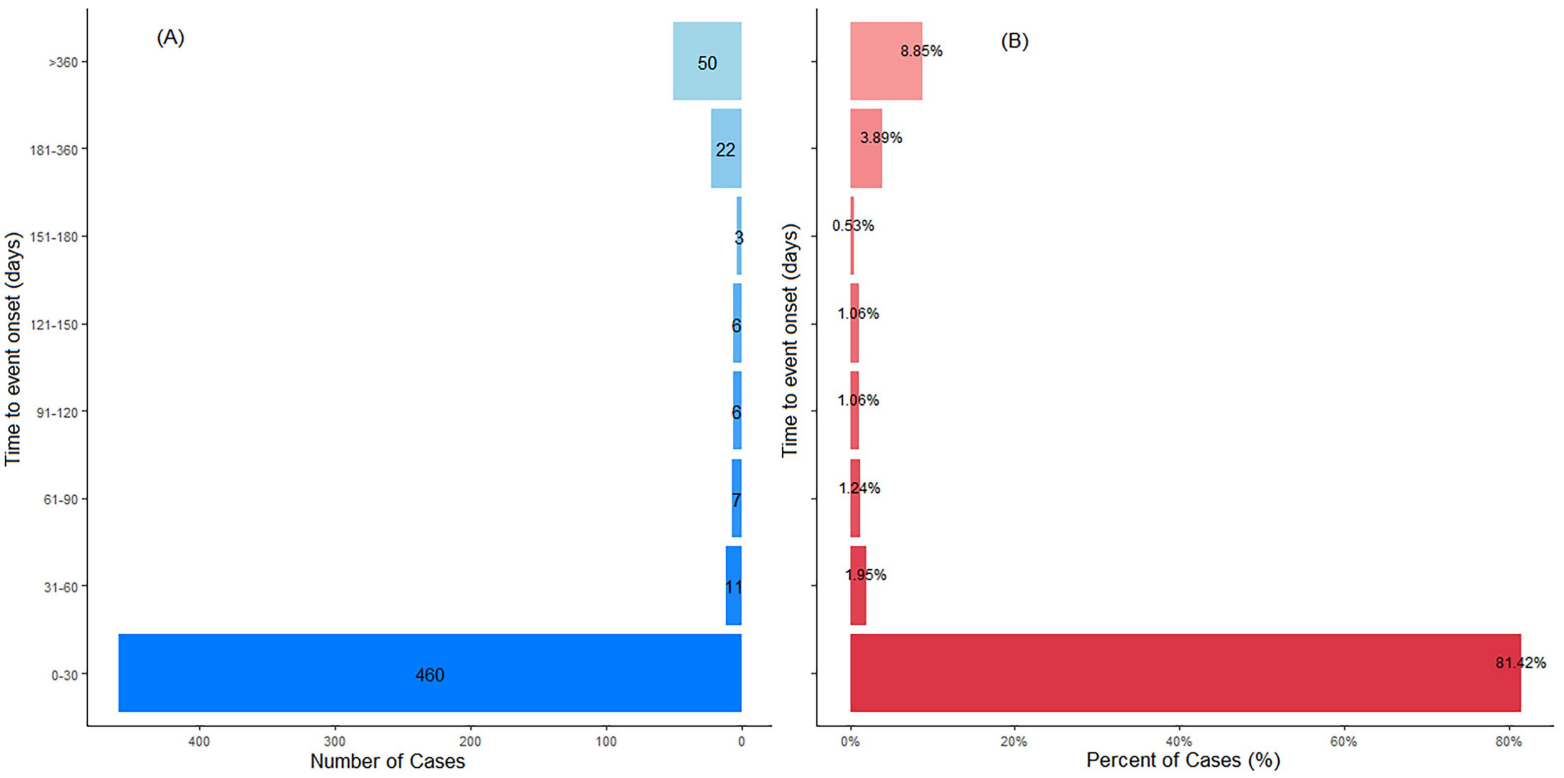
Temporal distribution and cumulative incidence of adverse events following Spiriva Respimat initiation. **(A)** Bar chart depicting the percentage of adverse event reports (*n* = 565 with documented time-to-onset data, representing 14.3% of total 3,962 reports) occurring within specified time intervals after treatment initiation. The majority of events (81.4%) occurred within the first 30 days, predominantly respiratory and gastrointestinal in nature. **(B)** Percentage distribution demonstrating the biphasic pattern with early peak (0–30 days) followed by late-onset tail (>360 days) primarily involving ocular and musculoskeletal systems. AEs, adverse events.

**Figure 3:**
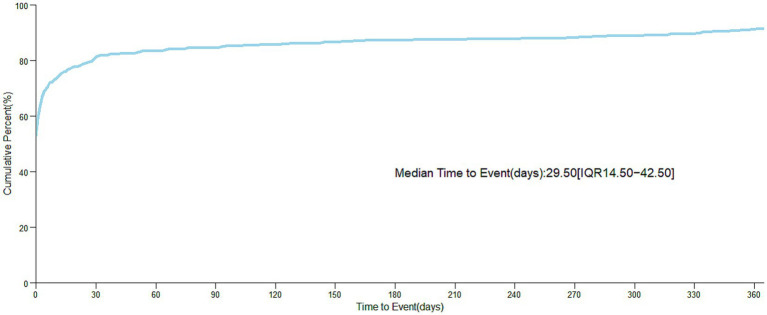
Kaplan–Meier curve of cumulative incidence for adverse events associated with Spiriva Respimat. The curve depicts the cumulative incidence of adverse events over time following drug administration, based on 565 reports with available time-to-onset data. The median time to onset was 29.5 days (interquartile range [IQR], 14.50–42.50 days), indicated by the solid vertical line. Dashed vertical lines represent the 25th and 75th percentiles. The rapid increase within the first month plateaus after 30 days, followed by gradual accumulation of late-onset events beyond 360 days. IQR, interquartile range.

Analysis of 565 reports with documented time-to-onset data (14.3% of total 3,962 reports) revealed distinct temporal patterns with important implications for monitoring strategies (Figures 2, 3). We acknowledge that the limited availability of time-to-onset data is a potential source of bias; reports with missing onset timing may not be randomly distributed across adverse event types. To assess whether this could affect conclusions, we compared the demographic characteristics and SOC-level adverse event frequencies between reports with and without timing data. No significant differences were observed in sex distribution, reporter type, or top-ranked SOC categories, suggesting that timing-documented reports are broadly representative of the full cohort. Nevertheless, the temporal patterns presented should be considered hypothesis-generating and interpreted with appropriate caution.

#### 3.4.1 Early-onset events (0–30 days): 81.4% of total

*Median onset*: 29.5 days (IQR: 14.5–42.5 days).

*Predominant types*: Respiratory (68%) and gastrointestinal (22%) events.

*Clinical interpretation*: These early events likely represent direct pharmacological effects, improper inhaler technique, or patient intolerance.

*Monitoring recommendation*: Close follow-up within the first month, particularly weeks 2–4, with assessment of symptom response and adverse effects.

#### 3.4.2 Late-onset events (>360 days): 8.9% of total

*Median onset*: 507 days (IQR: 422.5–674.5 days).

*Predominant types*: Ocular (41%) and musculoskeletal (33%) disorders.

*Clinical interpretation*: Delayed onset suggests cumulative or chronic exposure effects rather than acute pharmacological reactions.

*Monitoring recommendation*: Annual ophthalmologic examinations and periodic bone health assessment in long-term users, especially those >65 years.

#### 3.4.3 Intermediate events (30–360 days): 9.7% of total

Mixed pattern including infections, device-related issues, and persistent anticholinergic effects. Figure 2 displays the time distribution, highlighting the biphasic pattern with an early peak and late tail. Figure 3 presents the cumulative incidence curve, demonstrating rapid accumulation in the first month followed by gradual increase thereafter.

*Clinical takeaway*: This temporal analysis supports a risk-stratified monitoring approach: intensive early surveillance (0–30 days) for acute adverse effects, followed by annual long-term monitoring for delayed complications in chronic users.

## 4 Discussion

### 4.1 Principal findings and clinical implications

This comprehensive 20-year pharmacovigilance analysis of 3,962 adverse event reports provides important insights into the real-world safety profile of Spiriva Respimat with direct implications for clinical practice. While confirming established anticholinergic effects and respiratory adverse events, our study identifies three novel safety signals requiring heightened clinical attention:

1. Ocular complications (glaucoma and cataracts) with strong signal strength (ROR > 4.9) and delayed onset (median 507 days; IQR: 422.5–674.5 days), suggesting cumulative risk with chronic use.
2. Urogenital disorders (urinary retention) with very strong signal (ROR 6.01), particularly relevant for elderly males and those on concurrent anticholinergic medications.
3. Musculoskeletal events (fractures) with moderate signal strength, though confounding by corticosteroid use and disease-related frailty cannot be excluded.

The distinct temporal pattern—with 81.4% of events occurring within 30 days versus late-onset complications emerging after 12 months (>360 days)—supports a risk-stratified monitoring approach tailored to the exposure duration. These findings challenge clinicians to balance the well-established respiratory benefits of tiotropium against these underrecognized risks, particularly in vulnerable populations.

### 4.2 Novel safety signals: clinical significance and management

Our analysis identified three categories of adverse events requiring heightened clinical attention beyond established anticholinergic effects.

#### 4.2.1 Ocular complications

Significant signals emerged for glaucoma (ROR 5.67, 95% CI 4.89–6.58) and cataracts (ROR 4.92, 95% CI 4.25–5.70), with late onset (median time-to-onset for overall ocular events: 278 days; specifically, 507 days (IQR: 422.5–674.5 days) for late-onset cases occurring after one year), suggesting cumulative risk with chronic use. Inhaled tiotropium can reach ocular tissues through systemic absorption or inadvertent eye exposure during administration. Muscarinic receptor blockade in the eye may increase intraocular pressure by impairing aqueous humor drainage (14) and alter lens homeostasis through oxidative stress mechanisms (15).

##### 4.2.1.1 Clinical management recommendations

Pre-treatment assessment should include ocular history (known glaucoma, elevated intraocular pressure, cataracts, family history) with baseline ophthalmologic examination considered for patients >65 years or those with risk factors. Patient education on proper inhaler technique to avoid eye exposure is essential, with instruction to wash eyes immediately if accidental spray contact occurs. Annual ophthalmologic examination is recommended for chronic users (>6 months), with earlier evaluation (3–6 months) if risk factors present including age >75 years, family history of glaucoma, pre-existing lens opacities, or diabetes mellitus, consistent with American Academy of Ophthalmology guidelines for glaucoma screening (16). For patients with established glaucoma (especially angle-closure type) or uncontrolled elevated intraocular pressure, alternative LAMAs with potentially lower systemic absorption should be considered.

#### 4.2.2 Urogenital disorders

Urinary retention (ROR 6.01, 95% CI 5.32–6.79) was among the strongest signals, reflecting systemic anticholinergic effects particularly relevant given the elderly COPD population’s high prevalence of benign prostatic hyperplasia (BPH), affecting over 50% of men aged 60–69 years (17). High-risk patients include males >70 years with lower urinary tract symptoms (LUTS), International Prostate Symptom Score (IPSS) > 15, post-void residual >100 mL, concurrent anticholinergic medications (overactive bladder agents, antihistamines, tricyclic antidepressants), and history of urinary retention.

##### 4.2.2.1 Clinical assessment and monitoring

Pre-treatment screening should include standardized questions about urinary symptoms, with International Prostate Symptom Score (IPSS) questionnaire for males >70 years or those with LUTS, following AUA/SUFU guideline recommendations (17). Risk-stratified management includes: for IPSS <7 and post-void residual <50 mL, proceed with standard monitoring; for IPSS 8–19 or post-void residual 50–100 mL, use caution with enhanced 2-week follow-up; and for IPSS ≥20 or post-void residual >100 mL, strongly prefer alternative LAMA or LABA therapy. Two-week follow-up after initiation should reassess voiding symptoms, with patient education on warning signs including inability to urinate, weak stream, or straining to empty bladder. If retention occurs, immediately discontinue Spiriva Respimat, assess for urologic emergency, review all anticholinergic medications for deprescribing opportunities, and switch to alternative bronchodilator (LABA or alternative LAMA such as glycopyrronium or umeclidinium).

#### 4.2.3 Musculoskeletal events

Fracture signals (4.8% of reports, vertebral ROR 3.12, femoral ROR 3.45) require cautious interpretation due to substantial confounding. Contributing factors include inhaled corticosteroid use (70–80% of moderate–severe COPD patients) (18), COPD-related factors (sarcopenia, reduced physical activity, vitamin D deficiency, systemic inflammation affecting bone metabolism) (19, 20), smoking history (which independently reduces bone mineral density and is nearly universal in COPD patients), underlying osteoporosis (estimated prevalence 35–60% in COPD populations, substantially higher than the general age-matched population), and demographic factors (female predominance 62.3%, age >65 years 38.2%). Because FAERS does not capture structured data on smoking status, bone mineral density, or osteoporosis diagnosis at the case level, disproportionality analysis cannot isolate a direct tiotropium effect from these co-occurring risk factors. Direct anticholinergic effects on bone remain controversial (18), and our data cannot establish causation.

##### 4.2.3.1 Practical clinical approach

Rather than attributing fractures to tiotropium, implement comprehensive bone health assessment for all COPD patients. Calculate FRAX score to estimate 10-year fracture probability, with DEXA scan consideration if high risk, prior fragility fracture, or chronic corticosteroid use (>7.5 mg prednisone equivalent >3 months) (8). Risk mitigation includes calcium 1,200 mg/day plus vitamin D 800–1,000 IU/day, bisphosphonates for osteoporosis (T-score ≤ − 2.5), fall prevention strategies, and minimizing corticosteroid exposure. The fracture signal should not preclude tiotropium use but rather prompt integrated bone health management as part of holistic COPD care.

### 4.3 Clinical implications and risk management

#### 4.3.1 Risk stratification framework

To optimize benefit–risk balance, we propose systematic patient stratification. Low-risk patients (age <65 years, no significant comorbidities, no anticholinergic co-medications) receive standard monitoring with annual comprehensive assessment. Moderate-risk patients (age 65–80 years, ≤2 anticholinergic medications, mild LUTS or glaucoma suspicion) require baseline screening (ocular history, IPSS for males, FRAX score), 2-week and 4–6 week follow-up, and annual specialized assessments. High-risk patients (age >80 years, ≥3 anticholinergic agents, established glaucoma, BPH with IPSS >19, osteoporosis with prior fracture) warrant serious consideration of alternative LAMAs (glycopyrronium, umeclidinium) or non-LAMA options (LABA monotherapy, LABA/ICS), with intensive monitoring if Spiriva Respimat prescribed.

#### 4.3.2 Phased monitoring protocol

Our temporal analysis demonstrating 81.4% of events within 30 days versus late-onset complications at median 507 days (IQR: 422.5–674.5 days) supports phase-specific surveillance. Early monitoring (0–3 months) includes week 2 follow-up assessing respiratory status and adverse effects systematically (urinary symptoms, ocular symptoms, inhaler technique), week 4 in-person visit with comprehensive respiratory assessment and therapy continuation decision, and week 12 comprehensive evaluation transitioning stable patients to maintenance phase. Maintenance phase (3–12 months) involves quarterly follow-up monitoring respiratory control, adverse effects (particularly gradual onset urinary or visual symptoms), medication changes including new anticholinergic additions, and adherence assessment (3, 4). Long-term surveillance (annual for chronic users >12 months) targets late-onset complications with annual ophthalmologic examination for moderate-high risk patients, annual IPSS assessment for males with urinary risk, DEXA scan if indicated for fracture risk, and comprehensive anticholinergic burden reassessment.

#### 4.3.3 Shared decision-making

For moderate-to-high risk patients, structured discussion should cover benefits (reduces COPD exacerbations by ~20%, improves lung function and symptoms, confirmed in multiple trials) (1, 2, 11, 21), risks (10–20% chance of specific complications given patient’s risk factors), alternatives (glycopyrronium, umeclidinium, LABA options), monitoring plan, and patient values regarding trade-offs. Documentation should include discussion points, patient choice and rationale, and agreed monitoring plan. Detailed monitoring tools and patient education materials are recommended as components of routine clinical practice for this patient population.

### 4.4 Comparison with clinical trial data and other real-world studies

The TIOSPIR trial (*n* = 17,135) demonstrated non-inferiority of Respimat versus HandiHaler for mortality and exacerbations (11), but adverse event rates differed from our real-world findings. Dry mouth occurred in 2.8% (TIOSPIR) versus 9.1% (our study), while glaucoma, cataracts, and urinary retention were not separately itemized or reported as rare. These discrepancies reflect fundamental differences: clinical trials employ standardized adverse event collection with scheduled assessments while spontaneous reporting relies on voluntary recognition; trial populations systematically exclude very elderly (mean age 64–65 years in TIOSPIR versus 38% > 65 years in our reports) and those with significant comorbidities (7); and trial durations (median 2.3 years in TIOSPIR) may miss late-onset signals (median 507 days in our analysis) (11, 21).

Verhamme et al. (9) compared Respimat versus HandiHaler in European databases (*n* = 30,075) and found no mortality difference (HR 1.06, 95% CI 0.85–1.32) (9), corroborating TIOSPIR’s cardiovascular safety but not evaluating organ-specific adverse events beyond cardiopulmonary. Additional real-world safety studies from Italy (10) and East Asia (22) have further confirmed cardiovascular safety in diverse populations. Miravitlles et al. (7) demonstrated real-world patients are older, more comorbid, and on more medications than trial populations (7), supporting our hypothesis that vulnerable groups experience different adverse event profiles.

Our study uniquely contributes by characterizing long-term safety beyond typical trial durations, identifying ocular and urogenital signals not prominent in previous literature, defining temporal patterns informing monitoring strategies, and analyzing large, diverse real-world sample (*n* = 3,962 over 20 years) including vulnerable subgroups.

### 4.5 Innovative contributions of this study

Compared with prior FAERS-based pharmacovigilance studies of tiotropium, the present analysis offers several novel contributions. First, whereas previous real-world studies focused primarily on cardiovascular outcomes (9, 10), we systematically characterized adverse events across all 27 MedDRA organ system classes, revealing clinically significant signals in ocular, urogenital, and musculoskeletal domains that have received limited attention. Second, the integration of Weibull temporal modeling provides, to our knowledge, the first quantitative characterization of time-to-onset patterns for Spiriva Respimat adverse events, identifying a biphasic risk distribution (81.4% early-onset within 30 days vs. late-onset complications at median 507 days (IQR: 422.5–674.5 days) for events occurring >360 days, including ocular and skeletal complications) that has direct implications for monitoring frequency and timing. Third, compared with TIOSPIR (mean age 64–65 years, exclusion of severe comorbidities) (11), our real-world cohort enriches the evidence base for vulnerable populations (38.2% aged >65 years), in whom glaucoma and urinary retention carry particular clinical significance. These differences in adverse event rates between our study and TIOSPIR—such as dry mouth (9.1% vs. 2.8%) and the absence of itemized ocular/urogenital reporting in the trial—likely reflect differences in population vulnerability, voluntary reporting patterns, and the ability of passive surveillance to capture rare or gradual-onset events.

These signals are hypothesis-generating, requiring confirmation in controlled studies, but inform prudent risk management for high-risk populations pending definitive evidence (9).

### 4.6 Strengths, limitations, and future research

Study Strengths include large sample size (*n* = 3,962) over 20 years providing robust signal detection, multiple complementary methods (ROR, PRR, BCPNN, MGPS) enhancing confidence, temporal pattern analysis uniquely characterizing time-course, clinical focus translating findings into actionable recommendations, and transparent reporting of limitations.

FAERS Limitations are well-recognized. Data quality issues include missing information (age 42.9%, precise onset timing 14.3%) and reporter variability. The predominance of consumer-submitted reports (85.5%) warrants specific consideration: consumer reports may introduce lower diagnostic precision for objectively confirmed diagnoses (e.g., glaucoma, fracture), potential overreporting of subjective symptoms (e.g., dyspnea, dry mouth), and notoriety bias following media or regulatory attention (8). In this study, a formal sensitivity analysis restricted to healthcare professional reports (*n* = 563, 14.2% of total) was not conducted due to the substantially reduced sample size, which may limit the statistical power to detect rarer signals in this subset. This represents a study limitation, and future research should prospectively compare signal profiles between different reporter types to further characterize this potential bias in FAERS-based pharmacovigilance.

Causation cannot be established—only temporal association and reporting patterns suggesting possible links. Substantial confounding exists disease progression (COPD exacerbation, pneumonia), concomitant medications (ICS confounding fracture signal, other anticholinergics confounding urinary retention), and comorbidities (elderly baseline rates of cataracts, glaucoma, urinary symptoms). Reporting biases include stimulated reporting after regulatory warnings, underreporting of mild events, and notoriety bias for scrutinized drugs (8). Inability to calculate true incidence due to absent denominator data (total exposure person-years) limits absolute risk assessment—we can only evaluate disproportionality. Population representativeness is limited (US 85.1%, age underrepresentation, missing racial/ethnic data) (7, 9, 22). Dose–response information was not available for analysis in this study. The FAERS database records drug dose and duration in free-text fields that are inconsistently completed; in our dataset, structured dose information was missing in the majority of reports, precluding a formal dose–response analysis. Spiriva Respimat is approved at 2.5 μg (asthma) and 5 μg (COPD) doses (6, 13), and whether adverse event risk differs between these doses cannot be determined from our data. This represents an important direction for future investigation, ideally using electronic health records or claims databases that capture prescribed dose and cumulative exposure duration alongside adverse event outcomes.

Mitigation Strategies included rigorous deduplication, clinical review of implausible reports, sensitivity analyses, and clinical interpretation considering biological plausibility (e.g., anticholinergic mechanism supporting ocular and urinary signals) (14, 15, 17, 23), temporal relationships, and literature corroboration. We clearly state findings are hypothesis-generating associations requiring confirmation through observational studies with appropriate controls, following best practices for pharmacovigilance research (8, 24).

Based on the specific limitations and findings of this study, we propose the following targeted research priorities. (1) To address missing age data and confounding: future studies should leverage linked administrative databases (e.g., FAERS-Medicare linkage, UK Clinical Practice Research Datalink) that provide complete demographic and concomitant medication records, enabling propensity-score–adjusted comparisons between Spiriva Respimat and alternative LAMAs (glycopyrronium, umeclidinium) for ocular and urogenital outcomes. (2) To clarify dose–response relationships: prospective cohort studies or analyses of claims databases with structured dose fields should examine whether the 5 μg COPD dose confers greater risk of glaucoma or urinary retention than the 2.5 μg asthma dose, and whether cumulative exposure duration independently predicts late-onset complications. (3) To confirm ocular signals: ophthalmologic registry studies with baseline and serial intraocular pressure measurements in long-term Spiriva Respimat users (≥6 months) are needed to establish true incidence rates and confirm the causal direction of the glaucoma signal. (4) To resolve the fracture confounding: controlled observational studies using propensity-score matching should adjust specifically for concurrent inhaled corticosteroid dose, systemic corticosteroid exposure, and baseline bone mineral density to determine whether tiotropium contributes independently to fracture risk. (5) To evaluate concomitant LAMA effects: mechanistic pharmacokinetic studies should quantify systemic anticholinergic burden when Spiriva Respimat is co-administered with other anticholinergic agents, particularly in elderly patients with high baseline anticholinergic load. Long-term safety data in diverse populations, including those with renal impairment, remain limited and warrant dedicated investigation (25).

## 5 Conclusions and clinical recommendations

This comprehensive pharmacovigilance analysis of 3,962 adverse event reports over 20 years provides important insights into Spiriva Respimat’s real-world safety profile. While confirming established anticholinergic effects, we identified three novel signals requiring clinical attention: ocular complications (glaucoma, cataracts) with late onset (median 507 days; IQR: 422.5–674.5 days), urogenital disorders (urinary retention) with strong signal (ROR 6.01) particularly in elderly males with BPH, and musculoskeletal events (fractures) requiring integrated bone health assessment though causation remains uncertain due to confounding.

The distinct temporal pattern—81.4% of events within 30 days versus late complications at median 507 days (IQR: 422.5–674.5 days)—supports phase-specific monitoring: intensive early surveillance (weeks 0, 2, 4, 12) for acute effects and long-term annual monitoring (ophthalmology, urologic, bone health assessments) for chronic users.

### 5.1 Clinical action items

Before prescribing, complete systematic risk stratification categorizing patients as low, moderate, or high risk; conduct baseline assessments including ocular history and visual acuity for patients >65 years, IPSS questionnaire for males >70 years, and FRAX score for osteoporosis risk; consider alternatives for high-risk patients; and provide comprehensive patient education. During treatment, implement phased monitoring with early intensive surveillance, quarterly maintenance assessments, and annual long-term evaluations. High-priority monitoring includes annual ophthalmologic examination for chronic users (16), urinary symptom assessment for elderly males or those on multiple anticholinergics (17), and bone health evaluation for postmenopausal women and corticosteroid users (18).

### 5.2 Balancing benefits and risks

Spiriva Respimat remains valuable therapy with well-established benefits demonstrated in multiple large-scale trials (1, 2, 11–13, 21). These safety findings should inform patient selection and monitoring but not discourage appropriate use. Most patients—particularly low-to-moderate risk individuals—can safely use this medication with standard monitoring. For vulnerable populations, enhanced vigilance prevents serious complications. The proposed framework provides practical, evidence-based guidance for personalized prescribing that maximizes benefit while minimizing harm, drawing on both trial and real-world evidence (7, 9, 10, 22).

### 5.3 Practice transformation

We advocate shifting from “prescribe and monitor reactively” to “risk-stratify, personalize therapy, and implement targeted surveillance proactively.” By integrating systematic risk assessment, shared decision-making, and phased monitoring into routine respiratory care with attention to proper inhaler technique (3, 4), clinicians can optimize patient safety while preserving access to this valuable therapeutic agent for millions who benefit worldwide. Future research will clarify causal relationships and refine recommendations, but the framework presented here provides evidence-based guidance for safe, effective use of Spiriva Respimat in diverse clinical populations, following transparent research principles (8, 24).

## Data Availability

Publicly available datasets were analyzed in this study. This data can be found at: the raw data analyzed in this study are available from the FDA Adverse Event Reporting System (FAERS) public dashboard: https://fis.fda.gov/extensions/FPD-QDE-FAERS/FPD-QDE-FAERS.html.
