# Supplementary material for: Real world pharmacovigilance study of FDA adverse event reporting system events for Spiriva Respimat
Source: Front Med (Lausanne). 2026 May 29;13:1802121. doi: 10.3389/fmed.2026.1802121 (PMC13260142; doi:10.3389/fmed.2026.1802121)
Supplement: Supplementary file 1 [file Data_Sheet_1.docx]

Supplementary Material

Supplementary Tables

Supplementary Table 1 Two-by-two contingency table for disproportionality analyses

|  | Target AEs | Other AEs | Total |
| --- | --- | --- | --- |
| Spiriva Respimat | a | b | a+b |
| Other drugs | c | d | c+d |
| Total | a+c | c+d | a+b+c+d |

Abbreviations: AEs, adverse events; a, the number of reports containing target AEs caused by Spiriva Respimat; b, the number of reports containing other AEs caused by Spiriva Respimat; c, the number of reports containing target AEs caused by other drugs; b, the number of reports containing other AEs caused by other drugs.

Supplementary Table 2 Five major algorithms used for signal detection.

| Spiriva Respimat | Equation | Criteria |
| --- | --- | --- |
| ROR | 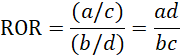  95%CI=e^ln(ROR)±1.96(1/a+1/b+1/c+1/d)^0.5^ | N≥3 and the 95% CI lower limit>1 |
| PRR | 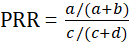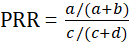  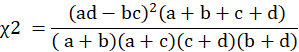 | PRR (95% CI): N≥3 and the 95% CI lower limit>1  PRR (χ^2^): N≥3, PRR≥2 and χ^2^≥4 |
| BCPNN | IC=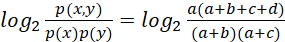  E(IC)=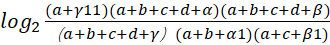  V(IC)=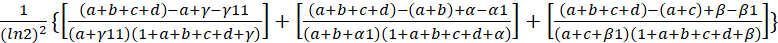  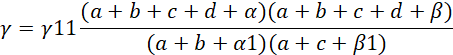  IC-2SD=E(IC)-2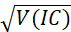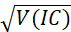  α1=β1, α=β=2 | Lower limit of IC025>0 |
| MGPS | 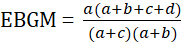  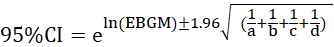 | EBGM05>2 |

Abbreviations: AEs, adverse events; a, the number of reports containing target AEs caused by Spiriva Respimat; b, the number of reports containing other AEs caused by Spiriva Respimat; c, the number of reports containing target AEs caused by other drugs; b, the number of reports containing other AEs caused by other drugs; CI, confidence interval; N, the number of reports; χ^2^, chi-squared; IC, information component; IC025, the lower limit of 95% CI of the IC; E(IC), the IC expectations; V(IC), the variance of IC; EBGM, empirical Bayesian geometric mean; EBGM05, the lower limit of 95% CI of EBGM.

Supplementary Table 3 Signal strength of AEs at the Preferred Term (PT) level ranked by FAERS data.

| **Preferred Term (PT)** | **Case reports** | **ROR (95%CI)** | **PRR (95%CI)** | **IC (IC025)** | **EBGM** |
| --- | --- | --- | --- | --- | --- |
| dyspnoea | 835 | 9.52(8.87,10.22) | 8.83(8.27,9.42) | 3.14(3.02) | 8.82 |
| off label use | 434 | 3.31(3,3.64) | 3.21(2.93,3.52) | 1.68(1.53) | 3.21 |
| cough | 353 | 7.92(7.12,8.8) | 7.68(6.93,8.51) | 2.94(2.75) | 7.67 |
| product quality issue | 314 | 13.77(12.3,15.41) | 13.38(12,14.92) | 3.74(3.52) | 13.35 |
| chronic obstructive pulmonary disease | 243 | 28.53(25.11,32.41) | 27.88(24.62,31.58) | 4.79(4.45) | 27.75 |
| asthma | 207 | 12.04(10.49,13.82) | 11.82(10.33,13.53) | 3.56(3.28) | 11.80 |
| pneumonia | 181 | 3.44(2.97,3.99) | 3.4(2.94,3.93) | 1.76(1.53) | 3.40 |
| wheezing | 139 | 14.86(12.57,17.58) | 14.68(12.44,17.32) | 3.87(3.49) | 14.64 |
| therapeutic product effect incomplete | 105 | 9.39(7.75,11.38) | 9.31(7.69,11.26) | 3.22(2.82) | 9.29 |
| chest discomfort | 87 | 5.21(4.22,6.43) | 5.17(4.19,6.38) | 2.37(1.99) | 5.17 |
| oropharyngeal pain | 86 | 5.61(4.54,6.94) | 5.57(4.52,6.88) | 2.48(2.09) | 5.57 |
| dry mouth | 85 | 6.4(5.17,7.92) | 6.35(5.14,7.85) | 2.67(2.27) | 6.35 |
| dysphonia | 72 | 7.45(5.91,9.4) | 7.41(5.88,9.32) | 2.89(2.43) | 7.40 |
| intentional product use issue | 71 | 5.04(3.99,6.36) | 5.01(3.97,6.31) | 2.32(1.9) | 5.00 |
| covid-19 | 71 | 2.39(1.89,3.01) | 2.38(1.89,3) | 1.25(0.88) | 2.38 |
| loss of personal independence in daily activities | 69 | 9.35(7.38,11.85) | 9.29(7.35,11.76) | 3.21(2.71) | 9.28 |
| device malfunction | 69 | 6.78(5.35,8.59) | 6.74(5.33,8.53) | 2.75(2.29) | 6.73 |
| extra dose administered | 64 | 11.31(8.84,14.46) | 11.24(8.8,14.36) | 3.49(2.92) | 11.22 |
| choking | 61 | 19.1(14.84,24.58) | 18.99(14.78,24.4) | 4.24(3.51) | 18.93 |
| lung neoplasm malignant | 59 | 8.28(6.41,10.7) | 8.24(6.39,10.63) | 3.04(2.5) | 8.23 |
| sleep disorder due to a general medical condition | 57 | 28.61(22.04,37.14) | 28.46(21.95,36.89) | 4.82(3.89) | 28.32 |
| throat irritation | 52 | 7.13(5.43,9.37) | 7.1(5.41,9.32) | 2.83(2.27) | 7.09 |
| productive cough | 52 | 6.86(5.22,9.01) | 6.83(5.2,8.95) | 2.77(2.22) | 6.82 |
| product delivery mechanism issue | 50 | 87.38(66.04,115.63) | 86.97(65.81,114.92) | 6.42(4.6) | 85.60 |
| device issue | 49 | 3.66(2.76,4.84) | 3.65(2.76,4.82) | 1.87(1.38) | 3.64 |
| blood count abnormal | 42 | 14.94(11.03,20.23) | 14.88(11,20.13) | 3.89(3.05) | 14.84 |
| lung disorder | 42 | 5.21(3.85,7.06) | 5.19(3.84,7.02) | 2.38(1.8) | 5.19 |
| influenza | 42 | 2.39(1.77,3.24) | 2.38(1.76,3.22) | 1.25(0.77) | 2.38 |
| oxygen saturation decreased | 37 | 4.11(2.98,5.68) | 4.1(2.97,5.66) | 2.04(1.45) | 4.10 |
| dyspnoea exertional | 36 | 5.84(4.21,8.1) | 5.82(4.2,8.07) | 2.54(1.89) | 5.82 |
| bronchitis | 35 | 2.76(1.98,3.85) | 2.75(1.98,3.83) | 1.46(0.91) | 2.75 |
| intentional product misuse | 35 | 2.43(1.74,3.38) | 2.42(1.74,3.37) | 1.28(0.74) | 2.42 |
| cataract | 107 | 4.92(4.25,5.70) | 4.88(4.22,5.65) | 2.29(1.98) | 4.87 |
| obstructive airways disorder | 30 | 16.13(11.27,23.09) | 16.09(11.25,23.01) | 4(2.91) | 16.04 |
| retching | 29 | 8.26(5.74,11.89) | 8.24(5.73,11.85) | 3.04(2.2) | 8.23 |
| rhinorrhoea | 28 | 2.66(1.84,3.86) | 2.66(1.84,3.85) | 1.41(0.79) | 2.66 |
| emphysema | 28 | 16.1(11.11,23.35) | 16.06(11.09,23.27) | 4(2.86) | 16.02 |
| oral discomfort | 28 | 11.93(8.23,17.3) | 11.9(8.22,17.24) | 3.57(2.57) | 11.88 |
| dry throat | 27 | 16.05(10.99,23.42) | 16.01(10.98,23.34) | 4(2.83) | 15.96 |
| secretion discharge | 27 | 14.04(9.62,20.5) | 14.01(9.61,20.43) | 3.8(2.71) | 13.98 |
| neoplasm malignant | 27 | 2.44(1.67,3.56) | 2.43(1.67,3.55) | 1.28(0.66) | 2.43 |
| candida infection | 27 | 10.23(7.01,14.92) | 10.2(7,14.87) | 3.35(2.39) | 10.18 |
| aphonia | 26 | 11.37(7.73,16.71) | 11.34(7.72,16.66) | 3.5(2.48) | 11.32 |
| product prescribing error | 26 | 7.05(4.8,10.37) | 7.04(4.79,10.34) | 2.81(1.97) | 7.03 |
| dose calculation error | 25 | 277.25(185.4,414.59) | 276.58(185.13,413.21) | 8.04(4) | 263.15 |
| nasal congestion | 25 | 2.64(1.78,3.91) | 2.64(1.78,3.9) | 1.4(0.74) | 2.63 |
| respiration abnormal | 25 | 22.89(15.44,33.91) | 22.83(15.42,33.8) | 4.51(3.06) | 22.74 |
| respiratory tract congestion | 22 | 8.46(5.57,12.86) | 8.45(5.56,12.83) | 3.08(2.07) | 8.44 |
| medication error | 21 | 2.28(1.49,3.5) | 2.28(1.49,3.5) | 1.19(0.49) | 2.28 |
| device delivery system issue | 20 | 6.6(4.25,10.23) | 6.59(4.25,10.21) | 2.72(1.75) | 6.58 |
| urinary retention | 155 | 6.01(5.32,6.79) | 5.96(5.28,6.73) | 2.58(2.28) | 5.95 |
| incorrect route of product administration | 17 | 6.68(4.15,10.75) | 6.67(4.15,10.73) | 2.74(1.66) | 6.66 |
| hypoacusis | 17 | 2.41(1.5,3.87) | 2.4(1.49,3.87) | 1.26(0.48) | 2.40 |
| pulmonary mass | 17 | 6.95(4.32,11.18) | 6.94(4.31,11.16) | 2.79(1.7) | 6.93 |
| product complaint | 16 | 4.25(2.6,6.94) | 4.24(2.6,6.92) | 2.08(1.13) | 4.24 |
| lower respiratory tract infection | 16 | 2.23(1.37,3.65) | 2.23(1.37,3.64) | 1.16(0.36) | 2.23 |
| respiratory disorder | 16 | 3.19(1.95,5.21) | 3.19(1.95,5.2) | 1.67(0.8) | 3.19 |
| dysuria | 15 | 2.41(1.45,3.99) | 2.4(1.45,3.99) | 1.27(0.42) | 2.40 |
| lung infection | 14 | 4.85(2.87,8.2) | 4.85(2.87,8.19) | 2.28(1.2) | 4.85 |
| product dispensing error | 14 | 4.27(2.52,7.21) | 4.26(2.52,7.19) | 2.09(1.06) | 4.26 |
| macular degeneration | 13 | 6.65(3.86,11.47) | 6.65(3.86,11.45) | 2.73(1.47) | 6.64 |
| hip fracture | 13 | 2.45(1.42,4.22) | 2.45(1.42,4.21) | 1.29(0.38) | 2.44 |
| product packaging quantity issue | 12 | 7.66(4.35,13.5) | 7.65(4.35,13.47) | 2.93(1.54) | 7.64 |
| rib fracture | 12 | 3.5(1.99,6.16) | 3.5(1.99,6.16) | 1.81(0.75) | 3.49 |
| asthmatic crisis | 12 | 19.41(11.01,34.23) | 19.39(11,34.17) | 4.27(2.2) | 19.33 |
| dementia | 12 | 2.68(1.52,4.72) | 2.68(1.52,4.71) | 1.42(0.44) | 2.67 |
| eosinophil count increased | 11 | 7.94(4.4,14.35) | 7.94(4.39,14.33) | 2.99(1.49) | 7.93 |
| throat tightness | 11 | 2.44(1.35,4.41) | 2.44(1.35,4.41) | 1.29(0.29) | 2.44 |
| haemoptysis | 11 | 2.32(1.28,4.19) | 2.32(1.28,4.19) | 1.21(0.23) | 2.32 |
| sneezing | 11 | 2.99(1.66,5.41) | 2.99(1.66,5.4) | 1.58(0.52) | 2.99 |
| oral pain | 11 | 2.88(1.59,5.2) | 2.88(1.59,5.19) | 1.52(0.48) | 2.88 |
| spinal fracture | 11 | 3.39(1.87,6.12) | 3.38(1.87,6.11) | 1.76(0.66) | 3.38 |
| eye disorder | 11 | 2.03(1.12,3.66) | 2.03(1.12,3.66) | 1.02(0.07) | 2.03 |
| respiratory tract infection | 11 | 2.63(1.46,4.76) | 2.63(1.46,4.75) | 1.4(0.38) | 2.63 |
| disability | 11 | 3.34(1.85,6.04) | 3.34(1.85,6.03) | 1.74(0.65) | 3.34 |
| limb injury | 10 | 2.24(1.21,4.17) | 2.24(1.21,4.17) | 1.16(0.14) | 2.24 |
| drug delivery system malfunction | 10 | 12.43(6.68,23.12) | 12.42(6.68,23.09) | 3.63(1.73) | 12.39 |
| laryngitis | 10 | 5.63(3.03,10.47) | 5.63(3.03,10.46) | 2.49(1.11) | 5.62 |
| glaucoma | 123 | 5.67(4.89,6.58) | 5.61(4.84,6.51) | 2.49(2.18) | 5.60 |
| upper-airway cough syndrome | 9 | 6.82(3.54,13.11) | 6.81(3.54,13.1) | 2.77(1.19) | 6.81 |
| joint injury | 9 | 2.51(1.31,4.83) | 2.51(1.31,4.83) | 1.33(0.21) | 2.51 |
| sputum discoloured | 9 | 5.75(2.99,11.06) | 5.75(2.99,11.05) | 2.52(1.05) | 5.74 |
| pulmonary congestion | 9 | 3.99(2.08,7.67) | 3.99(2.07,7.66) | 1.99(0.7) | 3.99 |
| abnormal loss of weight | 9 | 8.16(4.24,15.7) | 8.15(4.24,15.67) | 3.03(1.33) | 8.14 |
| product taste abnormal | 8 | 3.65(1.82,7.3) | 3.65(1.82,7.29) | 1.87(0.53) | 3.64 |
| nasal dryness | 8 | 7.68(3.84,15.37) | 7.68(3.84,15.35) | 2.94(1.18) | 7.67 |
| product physical issue | 8 | 2.53(1.26,5.05) | 2.53(1.26,5.05) | 1.34(0.15) | 2.52 |
| upper limb fracture | 8 | 2.34(1.17,4.68) | 2.34(1.17,4.67) | 1.23(0.06) | 2.34 |
| bronchial obstruction | 8 | 32.82(16.37,65.78) | 32.79(16.37,65.7) | 5.03(1.89) | 32.60 |
| device defective | 8 | 4.39(2.19,8.78) | 4.38(2.19,8.77) | 2.13(0.71) | 4.38 |
| drug delivery system issue | 8 | 30.76(15.35,61.65) | 30.74(15.35,61.57) | 4.93(1.87) | 30.57 |
| acute respiratory failure | 8 | 2.56(1.28,5.12) | 2.56(1.28,5.11) | 1.35(0.16) | 2.56 |
| multiple allergies | 8 | 6.2(3.1,12.4) | 6.19(3.1,12.39) | 2.63(1.01) | 6.19 |
| bronchiectasis | 8 | 7.58(3.79,15.17) | 7.57(3.79,15.15) | 2.92(1.17) | 7.57 |
| pulmonary function test decreased | 7 | 9.37(4.46,19.67) | 9.36(4.46,19.64) | 3.22(1.17) | 9.35 |
| pneumothorax | 7 | 2.66(1.27,5.57) | 2.65(1.27,5.57) | 1.41(0.12) | 2.65 |
| blood test abnormal | 7 | 3.04(1.45,6.39) | 3.04(1.45,6.38) | 1.6(0.26) | 3.04 |
| rhinitis | 7 | 5.23(2.49,10.99) | 5.23(2.49,10.98) | 2.39(0.75) | 5.23 |
| intentional underdose | 7 | 5.78(2.75,12.14) | 5.78(2.75,12.12) | 2.53(0.83) | 5.77 |
| wrist fracture | 7 | 3.47(1.66,7.29) | 3.47(1.66,7.28) | 1.8(0.39) | 3.47 |
| aortic aneurysm | 7 | 5.3(2.52,11.12) | 5.3(2.52,11.11) | 2.4(0.76) | 5.29 |
| pulmonary pain | 7 | 15.38(7.32,32.31) | 15.37(7.32,32.28) | 3.94(1.44) | 15.33 |
| urine flow decreased | 6 | 14.84(6.66,33.08) | 14.83(6.66,33.04) | 3.89(1.22) | 14.79 |
| pharyngeal swelling | 6 | 4.19(1.88,9.33) | 4.19(1.88,9.32) | 2.07(0.43) | 4.19 |
| sinus congestion | 6 | 2.65(1.19,5.9) | 2.65(1.19,5.89) | 1.4(0.01) | 2.65 |
| asphyxia | 6 | 4.35(1.95,9.7) | 4.35(1.95,9.69) | 2.12(0.46) | 4.35 |
| choking sensation | 6 | 6.6(2.96,14.69) | 6.59(2.96,14.68) | 2.72(0.78) | 6.59 |
| oxygen consumption increased | 6 | 15.83(7.1,35.28) | 15.82(7.1,35.24) | 3.98(1.25) | 15.77 |
| sputum increased | 6 | 18.11(8.12,40.37) | 18.1(8.12,40.33) | 4.17(1.3) | 18.04 |
| pneumonia bacterial | 6 | 4.15(1.87,9.25) | 4.15(1.87,9.24) | 2.05(0.43) | 4.15 |
| pneumonia fungal | 6 | 9.93(4.46,22.12) | 9.92(4.45,22.09) | 3.31(1.03) | 9.90 |
| oxygen saturation abnormal | 6 | 14.02(6.29,31.26) | 14.02(6.29,31.23) | 3.81(1.2) | 13.98 |
| urinary hesitation | 6 | 14.45(6.48,32.21) | 14.44(6.48,32.17) | 3.85(1.21) | 14.41 |
| oral candidiasis | 6 | 2.85(1.28,6.34) | 2.85(1.28,6.34) | 1.51(0.08) | 2.85 |
| lip injury | 6 | 27.51(12.33,61.38) | 27.5(12.33,61.32) | 4.77(1.43) | 27.36 |
| dementia alzheimer's type | 6 | 3.82(1.72,8.51) | 3.82(1.72,8.5) | 1.93(0.35) | 3.82 |
| oxygen consumption decreased | 5 | 47.3(19.61,114.1) | 47.28(19.61,113.99) | 5.55(1.26) | 46.87 |
| frustration tolerance decreased | 5 | 3.87(1.61,9.3) | 3.87(1.61,9.29) | 1.95(0.21) | 3.87 |
| hand fracture | 5 | 4.23(1.76,10.17) | 4.23(1.76,10.16) | 2.08(0.28) | 4.23 |
| spinal compression fracture | 5 | 3.24(1.35,7.78) | 3.24(1.35,7.78) | 1.69(0.06) | 3.23 |
| glossitis | 5 | 8.34(3.47,20.04) | 8.33(3.47,20.03) | 3.06(0.73) | 8.32 |
| photopsia | 5 | 4.71(1.96,11.32) | 4.71(1.96,11.31) | 2.23(0.36) | 4.70 |
| pelvic fracture | 5 | 3.91(1.63,9.4) | 3.91(1.63,9.39) | 1.97(0.22) | 3.91 |
| wrong product administered | 5 | 4.93(2.05,11.86) | 4.93(2.05,11.85) | 2.3(0.4) | 4.93 |
| blood immunoglobulin e increased | 5 | 12.41(5.16,29.84) | 12.4(5.16,29.81) | 3.63(0.92) | 12.37 |
| lip haemorrhage | 5 | 23.34(9.69,56.2) | 23.33(9.69,56.15) | 4.54(1.12) | 23.23 |
| product counterfeit | 5 | 19.68(8.18,47.38) | 19.67(8.18,47.34) | 4.29(1.08) | 19.61 |
| lip disorder | 5 | 15.2(6.32,36.57) | 15.19(6.32,36.53) | 3.92(0.99) | 15.15 |
| device physical property issue | 5 | 4.88(2.03,11.73) | 4.88(2.03,11.72) | 2.28(0.39) | 4.87 |
| tongue discomfort | 5 | 7.34(3.05,17.66) | 7.34(3.05,17.64) | 2.87(0.66) | 7.33 |
| oxygen therapy | 4 | 16.68(6.25,44.52) | 16.68(6.25,44.49) | 4.06(0.72) | 16.63 |
| exercise tolerance decreased | 4 | 4.25(1.59,11.33) | 4.25(1.59,11.32) | 2.09(0.07) | 4.25 |
| vocal cord disorder | 4 | 11.9(4.46,31.74) | 11.89(4.46,31.71) | 3.57(0.61) | 11.87 |
| lung neoplasm | 4 | 5.28(1.98,14.09) | 5.28(1.98,14.08) | 2.4(0.22) | 5.28 |
| dependence on oxygen therapy | 4 | 56.11(20.95,150.3) | 56.09(20.95,150.19) | 5.8(0.93) | 55.53 |
| nosocomial infection | 4 | 10.51(3.94,28.03) | 10.5(3.94,28.01) | 3.39(0.56) | 10.49 |
| halo vision | 4 | 21.01(7.87,56.11) | 21.01(7.87,56.07) | 4.39(0.78) | 20.93 |
| monocyte count increased | 4 | 6.55(2.46,17.47) | 6.55(2.46,17.46) | 2.71(0.34) | 6.54 |
| intracranial aneurysm | 4 | 5.84(2.19,15.58) | 5.84(2.19,15.57) | 2.55(0.28) | 5.84 |
| carotid artery occlusion | 4 | 6.39(2.4,17.03) | 6.39(2.4,17.02) | 2.67(0.33) | 6.38 |
| lip pain | 4 | 8.29(3.11,22.11) | 8.29(3.11,22.09) | 3.05(0.46) | 8.28 |
| increased upper airway secretion | 4 | 4.38(1.64,11.67) | 4.37(1.64,11.66) | 2.13(0.09) | 4.37 |
| total lung capacity decreased | 3 | 11.41(3.67,35.42) | 11.41(3.67,35.4) | 3.51(0.22) | 11.38 |
| infective exacerbation of chronic obstructive airways disease | 3 | 26.05(8.38,80.99) | 26.04(8.38,80.95) | 4.7(0.4) | 25.92 |
| product packaging confusion | 3 | 7.28(2.35,22.61) | 7.28(2.35,22.59) | 2.86(0.06) | 7.27 |
| sinusitis fungal | 3 | 24.25(7.8,75.4) | 24.25(7.8,75.36) | 4.59(0.39) | 24.14 |
| increased bronchial secretion | 3 | 6.82(2.2,21.18) | 6.82(2.2,21.17) | 2.77(0.03) | 6.82 |
| lung carcinoma cell type unspecified recurrent | 3 | 30(9.64,93.33) | 29.99(9.64,93.28) | 4.9(0.42) | 29.83 |
| spirometry abnormal | 3 | 33.06(10.62,102.89) | 33.05(10.63,102.83) | 5.04(0.43) | 32.86 |
| mucosal hypertrophy | 3 | 137.3(43.64,431.91) | 137.26(43.65,431.64) | 7.06(0.52) | 133.88 |
| wrong dose | 3 | 7.84(2.53,24.34) | 7.84(2.53,24.32) | 2.97(0.09) | 7.83 |
| respiratory gas exchange disorder | 3 | 46.42(14.9,144.66) | 46.41(14.9,144.57) | 5.52(0.47) | 46.02 |
| nocturnal dyspnoea | 3 | 14.01(4.51,43.52) | 14.01(4.51,43.5) | 3.8(0.28) | 13.98 |
| drug effect variable | 3 | 14.32(4.61,44.49) | 14.32(4.61,44.46) | 3.84(0.28) | 14.29 |
| small cell lung cancer | 3 | 13.69(4.41,42.53) | 13.69(4.41,42.5) | 3.77(0.27) | 13.66 |
| pulmonary function test abnormal | 3 | 11.22(3.61,34.83) | 11.22(3.61,34.81) | 3.48(0.21) | 11.20 |
| respiratory tract irritation | 3 | 17.74(5.71,55.13) | 17.74(5.71,55.1) | 4.14(0.33) | 17.68 |
| respiratory fume inhalation disorder | 3 | 58.91(18.88,183.83) | 58.9(18.88,183.71) | 5.86(0.48) | 58.27 |
| bronchitis chronic | 3 | 7.09(2.28,21.99) | 7.09(2.28,21.98) | 2.82(0.05) | 7.08 |

Abbreviation: PT, preferred term; ROR, reporting odds ratio; CI, confidence interval; PRR, proportional reporting ratio; IC, information component; IC025, the lower limit of the 95% CI of the IC; EBGM, empirical Bayesian geometric mean. Signals are detected when all the following criteria are met: N≥3, PRR≥2 and Chi-Square (χ^2^) ≥4, lower limit of 95% CI of ROR>1, IC025>0, EBGM05>2.
